# Exploring Age-Related Patterns in Smartphone Keystroke Dynamics Considering Temporal Variability: Cross-Sectional Study With AI-Based Analysis

**DOI:** 10.2196/80094

**Published:** 2025-12-02

**Authors:** Junhyung Moon, Yu Lim Huh, Hee Young Cho, Chaeyeon Kim, Hyeongrae Lee, Dong Pyo Jang, Baek Hwan Cho

**Affiliations:** 1 Department of Biomedical Informatics, CHA University School of Medicine CHA University Seongnam Republic of Korea; 2 Department of Electronic Engineering Hanyang University Seoul Republic of Korea; 3 Department of Obstetrics and Gynecology, Seoul National University College of Medicine Seoul National University Hospital Seoul Republic of Korea; 4 Institute of Reproductive Medicine and Population, Medical Research Center Seoul National University Seoul Republic of Korea; 5 Department of Biomedical Engineering Hanyang University Seoul Republic of Korea

**Keywords:** keystroke dynamics, digital biomarkers, age-related behavior, passive sensing, mobile health, temporal variability, artificial intelligence, mobile phone

## Abstract

**Background:**

Keystroke dynamics on smartphones have emerged as a promising form of passive digital biomarker. While previous studies have explored their utility in several diseases and disorders, relatively few have examined how these dynamics change systematically with chronological age in the general population.

**Objective:**

This study aimed to investigate age-related patterns in mobile keystroke dynamics, with a particular focus on temporal variations throughout the day. By identifying behavioral signatures associated with different age groups, we further assess whether artificial intelligence–based models can accurately estimate chronological age using passively collected keystroke data.

**Methods:**

We conducted a field study involving 177 healthy adults in the Republic of Korea, collecting free-living smartphone typing logs over multiple weeks through a custom Android keyboard app (CodeRed Corp). For each keystroke, the app recorded press and release timestamps and key type, from which 43 behavioral features were extracted across categories of speed, frequency, and temporal variability. Weekly feature vectors were constructed at 3 temporal resolutions (6-hour intervals, daily, and weekly). In total, 8 artificial intelligence models, including random forest, TabNet, transformer, and long short-term memory, were trained with participant-wise 10-fold cross-validation. A custom loss function was introduced to reduce intraparticipant prediction variability. Descriptive statistics and ablation studies were conducted to assess behavioral trends and feature contributions.

**Results:**

The study included 177 participants (female: n=115; male: n=62) with a mean age of 28.8 (SD 11.1) years, all residing in the Republic of Korea. On average, data were collected for 25 weeks per participant, resulting in a dataset of more than 2.5 million typing sessions. Descriptive analysis revealed clear age-related differences. Younger participants typed faster and more frequently, while older participants showed slower and more variable typing. The long short-term memory model using the 6-hour interval median features achieved the best age estimation performance (mean absolute error 3.69 years, *R*^2^=0.71). When the customized loss function was applied, the model’s performance further improved to a mean absolute error of 3.60, with a reduction in intraparticipant variability in estimated ages by 7.8%. Notably, feature importance analysis suggested that the early morning (midnight to 6 AM) and late evening (6 PM to midnight) periods may carry more age-discriminative keystroke patterns.

**Conclusions:**

Our findings demonstrated that smartphone keystroke dynamics reflect age-sensitive behavioral patterns, particularly when analyzed with fine-grained temporal resolution. While the primary goal was not age estimation per se, the ability to model these patterns highlights the potential of keystroke dynamics as a passive, unobtrusive behavioral marker for age-related functional characteristics. These insights may inform future applications in digital health, such as age-sensitive personalization or early detection of age-related decline without requiring any active user input.

## Introduction

The rapid expansion of mobile health apps has enabled individuals to monitor and manage their health conveniently and continuously. However, these services often rely on users’ active engagement, such as logging symptoms or responding to surveys. This dependence on manual input can present a barrier to long-term use. Passive sensing techniques can offer a promising alternative in digital health care, as they enable unobtrusive and continuous health monitoring without requiring explicit user participation [1-4].

Among various passive sensing modalities, digital biomarkers derived from everyday interactions with smartphones have received increasing attention, such as GPS [5,6], biosignals [7,8], and motion sensor data [9,10]. One promising approach involves analyzing mobile keystroke dynamics—patterns such as typing speed, interkey delay, and error frequency during smartphone keyboard usage. These subtle signals are unobtrusively collected and can reflect changes in cognitive and motor function [11-13], offering a valuable source of information for health-related inference.

Keystroke dynamics have been explored in previous work for various health domains like mild cognitive impairment [14], multiple sclerosis [15,16], loneliness [17], bipolar disorder [18], and mental health [13,19]. Specifically, Vesel et al [13] examined how mood, age, and diurnal patterns affect mobile keystroke dynamics using hierarchical mixed-effects models. However, their primary focus was on mood symptomatology—treating keystroke features, such as typing speed and variability, as outcomes influenced by depression severity, age, and time of day. Similarly, Zulueta et al [18] estimated brain age from smartphone typing kinematics and analyzed prediction error in relation to bipolar disorder risk. However, their focus was on diagnostic group differences rather than exploring how keystroke patterns change with age in the general population.

In contrast, our study aimed to conduct an in-depth investigation of age-related variations in keystroke dynamics, with particular attention to temporal keystroke patterns across different age groups. Given that aging is often accompanied by gradual changes in motor and cognitive function, these shifts may be subtly reflected in how individuals type on their smartphones. Importantly, such functional changes are not exclusive to aging—they may also result from sudden health deterioration. For instance, in patients at risk of cognitive impairment, subtle slowing and increasing variability in keystroke patterns may serve as early indicators of decline. Beyond health care, the ability to continuously and passively detect such functional changes could also contribute to security-related monitoring purposes. For instance, keystroke dynamics could reveal anomalous behavior when a compromised account is accessed by someone whose typing rhythm deviates markedly from the legitimate user, thereby serving as a continuous authentication layer.

## Methods

### Data Collection

We conducted a field study with a total of 177 healthy adults in the Republic of Korea from September 2022 to September 2023. Participants were recruited through online university communities and campus bulletin boards. Inclusion criteria were male and female individuals between 14 and 69 years of age without any mental or physical disabilities, who were Android smartphone users. Exclusion criteria were (1) inability or unwillingness to follow the investigator’s instructions; (2) difficulty in communication or lack of cooperation with the study procedures; (3) explicit refusal to participate in the study; (4) previous diagnosis of any psychiatric or physical illness; and (5) for minors (aged 14-19 years), lack of consent from a parent or legal guardian, or cases where parental consent was provided but the participant did not assent to participate. Those interested submitted an online application form that included their age, sex, smartphone operating system, preferred keyboard layout, and contact information. Eligible individuals were contacted to schedule an online meeting, during which they received a detailed explanation of the study and provided informed consent electronically. Following consent, participants were guided through the installation process and instructed to use our custom keyboard app as their default keyboard in daily life. The study was conducted exclusively in smartphone environments, and other platforms, such as desktop keyboards, were not included. Our custom keyboard app, developed for the Android operating system (CodeRed Corp), supported QWERTY layouts for Korean, English, numbers, and special characters. It recorded timestamps for each key-pressing and key-releasing event, along with the type of key (eg, Korean, English, delete, and special character). Per participant, a log file was generated, in which each row contained the timestamp of key press, the timestamp of key release, and the type of the pressed key. These files were automatically and securely transmitted to our research server on a daily basis. Data were collected during participants’ natural smartphone usage over a multiple-week period, without task-specific constraints, to ensure ecological validity.

### Ethical Considerations

The study was approved by the Institutional Review Board of Hanyang University (HYUIRB-202207-003-04). All participants were provided with detailed information about the study, including its objectives, the procedures for data collection, the devices and mobile apps to be used, the types of data to be collected, and the subsequent processing of these data. They were clearly informed that participation was entirely voluntary and that they could withdraw from the study at any time without penalty. Written informed consent was obtained from all participants before enrollment. A modest financial incentive was offered as compensation for their time and effort. The entire collected dataset was deidentified.

### Data Preprocessing and Feature Extraction

**Figure 1:**
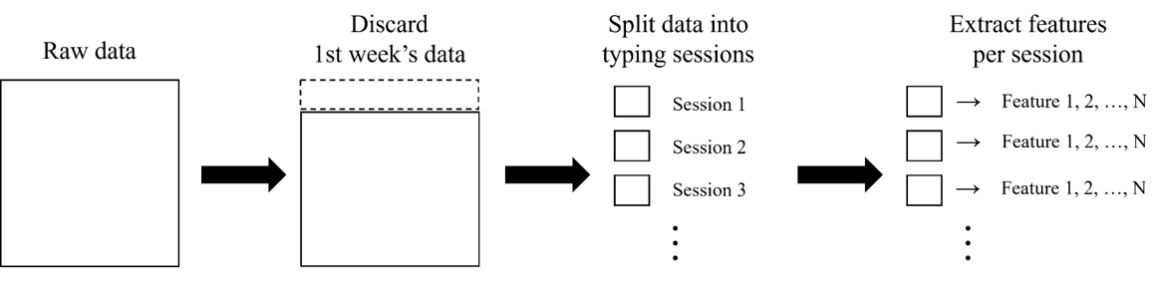
Flow of data preprocessing and feature extraction for each participant’s data.

As illustrated in Figure 1, we first discarded the data collected from each participant during the first week, since most participants spent the first week as an adaptation week to practice the provided keyboard app. For the remaining data, we excluded the key-press logs whose duration between press and release is longer than 5 seconds. Then, we processed the remaining logs in units of typing sessions. The typing session was determined by referring to the work by Vesel et al [13]: (1) duration between the keyboard activation and deactivation, or (2) if there was no press for more than 8 seconds after the last key release. We excluded the data of the typing sessions where key-pressing activities occurred more than or equal to 10 times per second (ie, interkey intervals ≤100 ms). This conservative threshold was chosen to eliminate abnormal or artifact-prone events, with reference to a large-scale study by Dhakal et al [20]. They analyzed 136,857,600 keystrokes from 168,960 participants who were asked to transcribe 15 English sentences using desktop keyboards. Their results showed that the average interkey interval across all participants was 239 milliseconds, whereas even the fastest typists exhibited an average of 122 milliseconds. Although their study was based on desktop keyboards, these findings provide a reasonable reference point for identifying abnormal keystrokes.

Within each typing session, we calculated speed and frequency-related features (speed and frequency, respectively, in Table 1), based on the existing studies [13,15-18]. The speed feature includes hold time (HT; a duration of each key press), flight time (a duration between release of the previous key and press of the current key), press-to-press latency (PPL), and release-to-release latency. Additionally, we calculated the correction, the HT for the delete key, and pre- and postcorrection, the flight times before and after pressing the delete key, respectively. The frequency features are the number of times any key is pressed and pressing the delete key, respectively (AllKey and DEL). In addition, we calculated various features in order to represent keystroke dynamics in terms of speed and frequency in a more delicate manner. First, we added the features regarding the keyboard layout shift (eg, from Korean QWERTY to English QWERTY): duration of layout shift (dur_shift) and the number of layout shifts (num_shift). Second, we counted the number of pressing any keys and the delete key per second, respectively (AllKeyRate and DELRate). Third, we added 2 features, that is, the duration of typing session (dur_session) and the number of typing sessions (num_session).

**Table 1:** Keystroke dynamics features (n=43) categorized into speed-related, frequency-related, and temporal variability-related features, which were used as input variables for the analyses in our study.

Category	Value, n	Features
Speed	11	HT^a^, FT^b^, PPL^c^, RRL^d^, precorrection, correction, postcorrection, AllKeyRate, DELRate, dur_shift, dur_session
Frequency	4	AllKey, DEL, num_shift, num_session
Speed variability	22	HT_MaxGap, FT_MaxGap, PPL_MaxGap, RRL_MaxGap, precorrection_MaxGap, correction_MaxGap, postcorrection_MaxGap, AllKeyRate_MaxGap, DELRate_MaxGap, dur_shift_MaxGap, dur_session_MaxGap, HT_STD, FT_STD, PPL_STD, RRL_STD, precorrection_STD, correction_STD, postcorrection_STD, AllKeyRate_STD, DELRate_STD, dur_shift_STD, dur_session_STD
Frequency variability	6	AllKey_MaxGap, DEL_MaxGap, num_shift_MaxGap, AllKey_STD, DEL_STD, num_shift_STD

^a^HT: hold time.

^b^FT: flight time.

^c^PPL: press-to-press latency.

^d^RRL: release-to-release latency.

Finally, we designed additional features to capture the temporal variability of speed and frequency features, respectively (speed variability and frequency variability in Table 1). We hypothesized that keystroke dynamics would fluctuate across different times of the day (eg, morning, afternoon, and night), analogous to the diurnal variations observed in physiological indicators [21]. We defined 2 types of variability features. The first, denoted as {Feature_Name}_MaxGap, represents the maximum fluctuation within a given time window (eg, day). It is calculated as the difference between the 95th and 5th percentiles of the feature’s values. The other is the SD of each feature’s values within a time window ({Feature_Name}_STD).

### Analysis Through Descriptive and Artificial Intelligence–Based Methods

We analyzed the extracted 43 features, as described in Table 1, across different chronological age groups (younger-than-30-years, 30s, 40s, and 50s). Considering potential options for the analysis time window, such as an hour, a day, and a week, we empirically determined to conduct an analysis on a weekly basis. We believed that a larger window, such as a month, might fail to capture the temporary change in health status, while a smaller window, such as a day, would lead to unnecessarily frequent analyses, introducing redundancy without improving interpretability. For visualization, we followed a three-step aggregation process. First, for each participant, multiple values of each feature were extracted within every 1-hour window. Second, the median value of each feature was computed per participant for each hour. Finally, the average of these per-participant medians was calculated across all participants within the same age group for each hour.

**Figure 2:**
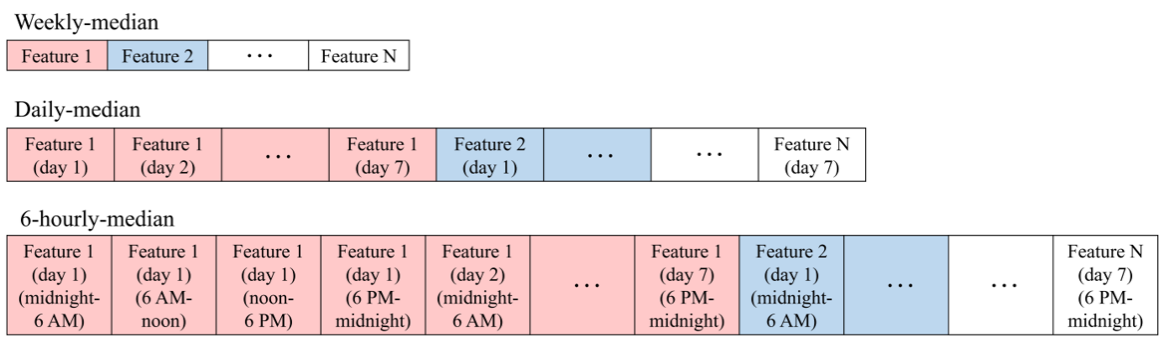
Examples of weekly feature vectors according to different temporal resolutions.

In addition to the descriptive analysis, we investigated the feasibility of artificial intelligence (AI)–based age estimation. We organized the extracted features as weekly feature vectors to serve as inputs for nonsequential and sequential AI models, introduced in the Age Estimation Model Development section. To construct the weekly feature vectors for nonsequential models, we used 3 temporal resolutions: weekly median, daily median, and 6-hour interval median (Figure 2). In the weekly median case, we built a feature vector using the median values of all features for each week. In the daily median case, we organized a feature vector by arranging the median value of each feature per day within each week. Considering the diurnal rhythm mentioned above, we split a day-resolution into hour levels. In the 6-hour interval median case, we constructed a feature vector by arranging the median value of each feature in 6-hour intervals (midnight-6 AM, 6 AM-noon, noon-6 PM, and 6 PM-midnight) for each week. For sequential models, such as a transformer-based and the long short-term memory (LSTM)–based models, the same features were organized as a temporal sequence across days and 6-hour periods, allowing the model to learn temporal dependencies. Accordingly, we used the daily median and 6-hour interval median resolutions only for sequential models, since the sequential information was already abstracted as a median value in the case of the weekly median. Thus, for sequential models, the feature vector using the daily median has a sequence length of 7, while the feature vector using the 6-hour interval median has a sequence length of 28. Given the importance of data order in sequential models, we further adjusted the organized feature vectors to begin with Monday’s data and end with Sunday’s data.

### Age Estimation Model Development

To train and evaluate AI models, we constructed 7 datasets from the original dataset to ensure balanced representation across different age groups. Specifically, we divided the data of participants younger than 30 years into 7 participant-wise subsets (17×1+18×6). This division was based on the average number of participants in the other 3 age groups (30s, 40s, and 50s), which was approximately 17.3. Thus, we used 17 and 18 as the subset sizes for the younger-than-30-years group. Each subset of the younger-than-30-years group was then combined with the full data from the other age groups to form 7 age-balanced datasets. The resulting datasets had mean ages ranging from 38.5 to 38.9 (SD 0.1) years. Sex distribution was also comparable across the datasets, with the number of female participants ranging from 41 to 48 and male participants from 22 to 29. Within each dataset, missing values were imputed using the values from the nearest week. Minimum-maximum normalization was applied within each constructed dataset.

For the age estimation in this study, we used 8 AI models: logistic regression, random forest regressor, extreme gradient boosting (XGBoost) regressor, CatBoost regressor, multilayer perceptron (MLP), and TabNet [22], as well as sequential models based on a transformer and LSTM. Using Python version 3.11.4, we implemented the 8 AI models, which take the constructed weekly feature vectors and then output the estimated age.

For each of the 7 age-balanced datasets, we conducted 10-fold cross-validation while exploring the hyperparameter space to maximize the regression performance. We divided the individual dataset into 10 folds in a participant-wise manner, ensuring that the same participant’s data did not appear in both training and test sets. The fold construction was based solely on participant separation without rebalancing age distribution across folds. For logistic regression, we opted for a consistent hyperparameter set, choosing “liblinear” as the solver and “L2” for regularization. For the random forest regressor, XGBoost regressor, and CatBoost regressor, we adjusted the number of estimators and the depth of each estimator, additionally changing the learning rate only for the XGBoost regressor and CatBoost regressor. For MLP, we stacked BatchNorm1d, Linear, ReLU, and Dropout layers using the torch package. Within the architecture, we adjusted the number of layers, the number of nodes in each layer, the number of epochs, and the batch size. We used a dropout rate of 0.3, a weight decay of 0.001, and the ReduceLROnPlateau scheduler for the learning rate. For TabNet, a deep learning model designed for tabular data, we used the pytorch_tabnet package. We adjusted the dimension of decision prediction layers, the dimension of the attentive transformer, the number of decision steps, the number of epochs, and batch size, while using the Adam optimizer and the ReduceLROnPlateau scheduler, from the torch package, for the learning rate. For the transformer-based model, we examined various options for the number of layers, the number of attention heads, the hidden dimension, the learning rate, and the batch size. For the LSTM-based model, we regulated values of the number of layers, the number of nodes in each layer, the learning rate, and the batch size. For the deep learning models, in each fold, we stored the model from the epoch that achieved the best performance on the validation set and used it for evaluation, rather than simply reporting the last epoch.

We hypothesized that if an individual is in good health, the estimated age would remain relatively stable across different weeks. Given that our participants were from a healthy population, we customized the loss function of the AI model to minimize deviations in estimated age within the same participant. The training process was divided into 2 phases. In the first phase, we constructed mini-batches such that each batch contained multiple weekly samples from the same participant. For each batch, we calculated the average L1 loss between the real and estimated ages across the participant’s weekly samples. If this average L1 loss exceeded an empirically defined threshold, a penalty term—computed as the average L1 loss multiplied by a weight—was added to the original loss. We experimented with threshold values of 1, 2, and 3, and weight values of 0.2, 0.4, 0.6, 0.8, and 1.0. In the second phase, training continued using the standard L1 loss without the additional penalty. We tuned the number of epochs for each phase to achieve optimal age estimation performance.

Upon completion of model training, we assessed age estimation performance based on mean absolute error (MAE) and *R*^2^. Each metric was then averaged across the results from the 7 age-balanced datasets to obtain the final evaluation scores.

## Results

### Participant Data Overview

Table 2 shows the characteristics of the dataset of the entire 177 participants, including a sex distribution of 115 females and 62 males. All of them were residents of the Republic of Korea. The participants had a mean age of 28.8 (SD 11.1) years, with a minimum of 19 years and a maximum of 58 years. The majority (n=125) were younger than 30 years old, while the remaining participants were distributed across the following age groups: 30-39 years (n=16), 40-49 years (n=19), and 50-59 years (n=17). On average, data were collected over a 25-week period per participant, ranging from a minimum of 4 weeks to a maximum of 41 weeks. In total, 2,597,692 typing sessions were collected, of which 89,219 (3.4%) were discarded because they contained at least 1 instance of ≥10 consecutive keystrokes occurring within 1 second.

**Table 2:** Demographic distribution of participants and the duration of keystroke data collection across all participants in our study.

Item		Value
Participants, n		177
Age (y), mean (SD)		28.8 (11.1)
**Total group age range (y), n**		
	<30	125
	30-39	16
	40-49	19
	50-59	17
Sex (female:male), n		115:62
Data collection weeks, mean (SD; minimum-maximum)		25.0 (13.3; 4-41)

### Descriptive Analysis Results for Different Age Groups

**Figure 3:**
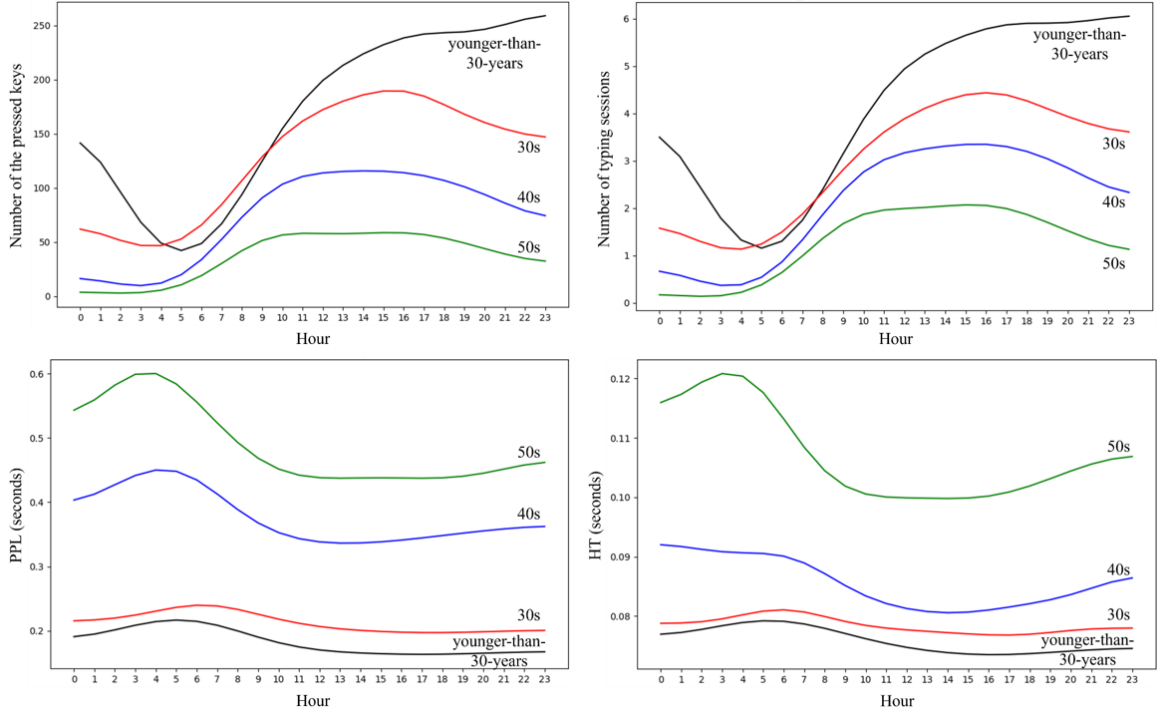
Visualization of keystroke dynamics across age groups, showing the hourly distribution of the number of key presses, the number of typing sessions, press-to-press latency, and hold time, highlighting age-related differences over the course of a day. HT: hold time; PPL: press-to-press latency.

Figure 3 illustrates the hourly patterns of 4 features—number of pressed keys, number of typing sessions, HT, and PPL—across different age groups (younger than 30 years, 30s, 40s, and 50s). As illustrated in Figure 3, the younger groups (younger than 30 years in black, and 30s in red) consistently exhibited a higher number of pressed keys throughout the day compared with the older groups (40s in blue, and 50s in green). Notably, the younger than 30 years group peaked at 11 PM, whereas the older groups showed a slight decline approaching that hour. The number of typing sessions showed a similar pattern. PPL remained low (approximately 0.2 seconds) for the younger groups, while it was higher for the older groups. HT followed a similar trend to PPL.

### Age Estimation Results Using Keystroke Dynamics

Table 3 presents the age estimation results obtained from the 8 AI models used in our study, based on speed and frequency features. These results were produced without applying the customized loss function, as our goal was to first report baseline performance from conventional AI models. Among the nonsequential models, MLP and TabNet demonstrated competitive performance, with MAEs of 3.85 and 4.01, respectively, under the weekly median resolution. These 2 deep learning–based models consistently outperformed traditional machine learning models across all temporal resolutions. Overall, the sequential model LSTM showed improved performance as the temporal resolution became finer. With the 6-hour interval median resolution, LSTM achieved an MAE of 3.69 and an *R*^2^ of 0.71, which is the optimal performance in this experiment. *R*^2^ generally increased with finer temporal resolutions across most of the 8 AI models. Although we also tested the 3-hour interval median resolution, it did not lead to further performance improvement (MAE remained at 3.69).

**Table 3:** Age estimation results of 8 artificial intelligence models using speed and frequency features and 3 different temporal resolutions (weekly median, daily median, and 6-hour interval median).

AI^a^ model			Weekly median					Daily median					6-hour interval median		
			MAE^b^		*R* ^2^		MAE			*R* ^2^		MAE			*R* ^2^
Nonsequential models															
	Logistic regression	6.43		0.01		6.77			–0.09		6.81			0.13	
	Random forest regressor	5.49		0.44		5.34			0.48		5.13			0.53	
	XGBoost^c^ regressor	5.54		0.47		5.38			0.49		5.07			0.55	
	CatBoost regressor	5.26		0.50		5.28			0.50		5.04			0.55	
	MLP^d^	3.85		0.69		4.19			0.65		4.36			0.65	
	TabNet	4.01		0.67		4.24			0.64		4.04			0.65	
Sequential models															
	Transformer	—^e^		—		4.51			0.59		4.56			0.58	
	LSTM^f^	—		—		3.86			0.68		3.69			0.71	

^a^AI: artificial intelligence.

^b^MAE: mean absolute error.

^c^XGBoost: extreme gradient boosting.

^d^MLP: multilayer perceptron.

^e^Not applicable.

^f^LSTM: long short-term memory.

### Impact of Different Combinations of Feature Sets

Using the LSTM model with the 6-hour interval median resolution, which achieved the optimal MAE of 3.69 (Table 3), we conducted an ablation study to examine the effectiveness of different feature set combinations in our age estimation task. For each experiment in Table 4, we regulated hyperparameters of the LSTM model to find the optimal MAE. Overall, the speed feature appeared more influential for age estimation than the frequency feature. Using only the speed feature yielded a comparable MAE of 3.70, in which both speed and frequency features were used (MAE=3.69). In contrast, the inclusion of speed and frequency variability features did not result in noticeable improvements, suggesting their limited effectiveness in this context. When used in isolation, the frequency feature and its variability yielded limited but marginal improvements.

**Table 4:** Results of the ablation analysis examining the impact of using different combinations of feature sets on age estimation performance.

Number of features	Speed	Frequency	Speed variability	Frequency variability	MAE^a^
15	✓	✓			3.69
43	✓	✓	✓	✓	3.88
11	✓				3.70
33	✓		✓		3.86
4		✓			6.33
10		✓		✓	5.89
22			✓		4.06
6				✓	6.37
28			✓	✓	4.07

^a^MAE: mean absolute error.

### Impact of Different Combinations of 6-Hour Periods

Using the LSTM model with the 6-hour interval median resolution, we conducted an additional ablation study to examine the impact of using different 6-hour periods within a single day. For this experiment, we fixed the hyperparameters to those of the optimal case presented in Table 3. As illustrated in Table 5, we conducted experiments using all possible combinations of the four 6-hour periods. Overall, the MAE increased as the total number of hours used for age estimation decreased. When using only one 6-hour period, the MAE ranged from 4.34 to 4.61, indicating relatively poor performance. With 12-hour combinations, the optimal performance (MAE=3.95) was achieved when using data from midnight-6 AM and 6 PM-midnight. A similar trend was observed for the 18-hour combinations, where the lowest MAE (3.87) occurred in 2 combinations that included these time intervals.

**Table 5:** Results of the ablation analysis examining the impact of using different combinations of 6-hour periods within a day on age estimation performance.

Total duration		Midnight-6 AM	6 AM-noon	Noon-6 PM	6 PM-midnight	MAE^a^
24 h						
	1	✓	✓	✓	✓	3.69
18 h						
	1		✓	✓	✓	4.02
	2	✓		✓	✓	3.91
	3	✓	✓		✓	3.87
	4	✓	✓	✓		3.87
12 h						
	1			✓	✓	4.13
	2		✓		✓	4.06
	3		✓	✓		4.26
	4	✓			✓	3.95
	5	✓		✓		3.96
	6	✓	✓			4.01
6 h						
	1	✓				4.43
	2		✓			4.61
	3			✓		4.50
	4				✓	4.34

^a^MAE: mean absolute error.

### Feature Selection and Optimized Estimation

We applied the previously introduced customized loss function to the LSTM model with the 6-hour interval median resolution to reduce intraparticipant deviation in estimated age. The model was trained from scratch and achieved an MAE of 3.60. To evaluate the effectiveness of the customized loss function, we computed the SD of estimated ages across weeks for each participant and then averaged these values across all participants. The average SD decreased from 1.28 (SD 0.22; without the loss function) to 1.18 (SD 0.14; with the loss function), indicating a 7.8% reduction in intraparticipant variability.

**Figure 4:**
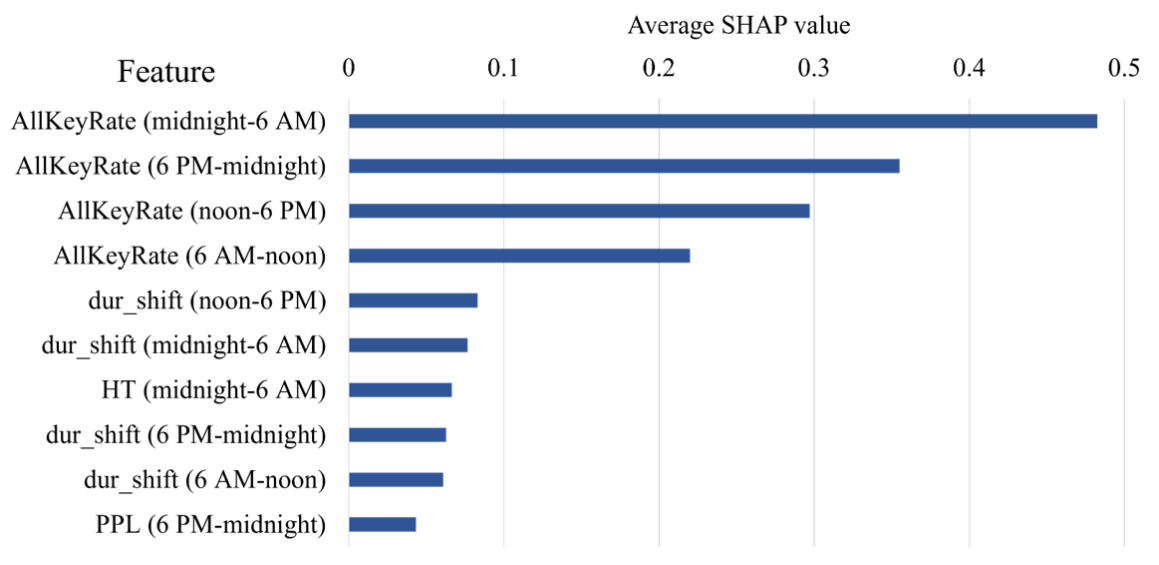
Top 10 most influential features for age estimation, identified via SHAP analysis of the long short-term memory model trained with the customized loss function and 6-hourly-median resolution. HT: hold time; PPL: press-to-press latency; SHAP: Shapley Additive Explanations.

We also applied Shapley Additive Explanations [23] to assess feature importance in the model trained with the customized loss function. As illustrated in Figure 4, the top 10 most influential features out of 60 were predominantly related to keystroke speed and rhythm, such as AllKeyRate, dur_shift, and HT, measured across various 6-hour time periods.

## Discussion

### Principal Results

This study aimed to investigate whether age-related differences manifest in everyday smartphone keystroke dynamics. Through both descriptive and AI-based analyses, we demonstrated that typing behavior—such as speed, latency, and session frequency—systematically varies across age groups. For instance, younger participants exhibited consistently faster and more frequent typing patterns, particularly during late evening hours, while older individuals showed slower and less variable patterns throughout the day.

Building on these observations, we explored whether such behavioral signals could support accurate AI-driven age estimation. Among the 8 models tested, the LSTM model with the 6-hour interval median resolution achieved the best performance (MAE=3.69, *R*^2^=0.71), highlighting that fine-grained temporal patterns carry meaningful age-related information. While predicted ages that differ from actual ages by only a small margin (eg, within ±5 y) may reflect normal variability, sudden large deviations (eg, 10-20 y) could arise from short-term factors, such as stress, fatigue, or sleep deprivation, as well as from long-term or chronic factors, such as disease onset or persistent cognitive or motor decline. Previous studies have shown that short-term conditions like fatigue or stress can alter keystroke dynamics, increasing interkey latencies and error rates [24,25]. Similarly, chronic neurological or cognitive disorders, including Parkinson disease and mild cognitive impairment, have been associated with measurable changes in typing patterns [26,27]. The magnitude of fluctuation in age predictions is therefore likely to depend on both the timescale and the underlying cause of behavioral change. Future work should not only disentangle short- and long-term contributors but also investigate how the scale of predicted age deviation corresponds to these different contributors. Such insights could enable adaptive age-prediction strategies that dynamically adjust to the likely source of variability, improving both robustness and interpretability in real-world conditions.

Ablation studies further revealed that typing speed features contributed most strongly to model performance, aligning with the behavioral differences observed in the descriptive analysis. Notably, our time-of-day ablation study (Table 5) revealed that the early morning (midnight-6 AM) and late evening (6 PM-midnight) periods contained more informative signals for age prediction. These findings suggest that age-related behavioral differences may be more pronounced during off-peak hours, potentially due to variations in daily routine structures, fatigue, or circadian rhythms.

Taken together, our results indicate that keystroke dynamics encode subtle, age-sensitive behavioral patterns that can be leveraged for passive, AI-based age estimation—without requiring any explicit input from users.

### Comparison With Previous Work

In total, 2 studies are particularly relevant for contextualizing our contributions, that is, studies by Vesel et al [13] and Zulueta et al [18].

Vesel et al [13] applied hierarchical mixed-effects models to examine how mood, age, and diurnal patterns influence keystroke behavior. They found that younger users (20 y) typed substantially faster, less variably, and paused less frequently compared with older users (≥70 y), with effect sizes ranging from 57% to 62%. Importantly, they also identified a significant interaction with diurnal patterns, such that older adults displayed greater slowing and variability in the later hours of the day. Consistent with these findings, our study also observed similar age-related differences in typing speed (Figure 3). However, Vesel et al [13] did not focus on prediction but rather on identifying how typing behavior varies as a function of demographic and clinical factors. They treated age as an independent variable affecting keystroke features, whereas in our study, age was the target variable to be predicted. Moreover, while Vesel et al [13] identified diurnal trends in typing speed and variability, our model leveraged this information to improve prediction through time-resolved feature representation and ablation experiments across 6-hour periods. In this sense, our study operationalizes the temporal behavioral patterns identified by Vesel et al [13] into an actionable, AI-driven predictive framework.

Zulueta et al [18], on the other hand, estimated “brain age” from mobile keystroke kinematics and examined the deviation between predicted and actual age in relation to bipolar disorder risk. They tokenized free-living iOS (BiAffect) typed logs into sessions and then summarized kinematic features per participant. To estimate chronological age, they trained random forest regressors on a 75:25 train-validation split using 10-fold cross-validation with 3 repeats. On the held-out validation set, they reported root mean square errors of 9.5-9.7 years, Breiman pseudo *R*^2^ of 0.42-0.44, and MAEs of 5.5-5.9 years. While they also performed age prediction using smartphone metadata, their goal was to assess brain age as a digital biomarker of psychiatric risk, particularly differentiating prediction error patterns across diagnostic groups (positive vs negative Mood Disorders Questionnaire screens). Their models were evaluated primarily using participant-level aggregates, whereas our study preserved temporal granularity by modeling week-by-week patterns within participants. We also proposed a novel loss function to explicitly reduce intraparticipant variability, which was not addressed in their work. Finally, our best-performing model achieved an MAE of 3.60, substantially lower than the 5.5-5.9 years reported by Zulueta et al [18], possibly indicating the advancement of our method.

In summary, while previous works have laid important theoretical foundations, our study offers a methodological advancement by combining fine-grained temporal feature engineering, intraparticipant variance penalization, and deep learning architectures for high-resolution, week-level age estimation.

### Limitations

Despite promising findings, our study has several limitations. First, our dataset was heavily skewed toward younger participants who were younger than 30 years of age. Although we constructed age-balanced subsets to partially mitigate this imbalance, the initial sampling bias may still have influenced model training and evaluation. In particular, residual imbalance across the cross-validation folds could not be fully eliminated, which may have led to performance estimates biased toward younger age groups. Future studies should validate our findings on larger and more demographically diverse populations, including a broader distribution of age groups.

Second, all participants in this study were healthy individuals without known motor or cognitive impairments. While this design choice helped isolate age as a primary factor, it limits the generalizability of our findings to clinical populations. Since keystroke dynamics are known to be affected by neurological and psychiatric conditions, future work should explore how such models perform across varying health statuses.

Third, our study population consisted exclusively of Korean Android users, which may limit the generalizability of our findings. Cultural factors (eg, language-specific typing conventions), linguistic aspects (such as Korean vs alphabet-based input), and operating system–specific characteristics (eg, Android vs iOS) could all affect keystroke dynamics. Furthermore, our study did not consider keystroke dynamics on desktop or other mobile devices (eg, tablet), which may also limit generalizability. To strengthen external validity, future studies should validate the model across multiple platforms and operating systems by including both Android and iOS users, expand recruitment to participants from different countries and linguistic backgrounds, and incorporate broader age groups, including younger adolescents and older adults. Such efforts would allow more comprehensive evaluation of the robustness and applicability of keystroke-based age prediction models.

Fourth, another limitation is that individual and device-specific factors, such as the dominant hand or screen size, were not considered in this study. These factors may systematically influence typing speed, rhythm, and error patterns, thereby introducing variability that is independent of age-related effects. For instance, larger screens may allow for more stable keystrokes, while different input languages and keyboard layouts could alter interkey intervals. Although such factors may add noise to the prediction, they are unlikely to fully explain the observed associations, yet future work should explicitly account for them to improve model validity and generalizability.

Fifth, our study did not evaluate the long-term temporal stability of the proposed models. Although we used multiweek data for training and testing, we did not assess whether age predictions remain consistent over several months. This is especially important for real-world applications where behavioral patterns may shift gradually due to lifestyle changes, seasonality, or device replacement.

Sixth, although our dataset volume was large, deeper statistical analysis revealed that the keystroke features did not follow Gaussian distributions. Specifically, we conducted Shapiro-Wilk normality tests across all features, and none satisfied the assumption of normality (all *P* values <.001). As a result, Gaussian-based methods, such as linear or quadratic discriminant analysis, would not have been appropriate for this dataset. This supports our choice of nonlinear AI-based approaches, which are better suited to capture the inherently non-Gaussian nature of keystroke dynamics.

Finally, while our analysis of temporal variance features (speed variability and frequency variability) did not lead to meaningful performance gains, we believe this area holds potential. The human body follows natural diurnal rhythms that affect motor and cognitive function across the day. With more refined feature engineering, temporal variability may better capture such rhythms and contribute to improved age estimation in future models.

### Conclusions

This study demonstrates that everyday smartphone keystroke dynamics contain measurable behavioral patterns that vary systematically across age groups. By analyzing typing speed, rhythm, and frequency features across different time windows, we showed that younger and older individuals exhibit distinct temporal keystroke signatures—with our results suggesting that early morning and late evening periods may carry more predictive information for age estimation. These findings suggest that keystroke dynamics may serve as meaningful digital behavioral markers of age-related functional differences.

Building on these behavioral insights, we developed AI models to estimate age using passive keystroke data, with the LSTM model and the 6-hour interval median resolution achieving the best performance (MAE=3.69, *R*^2^=0.71). We further introduced a customized loss function that reduced intraparticipant variability, and used Shapley Additive Explanations analysis to identify key typing features contributing to model predictions.

Importantly, this work is not merely an age estimation exercise. Rather, it illustrates how fine-grained, temporally structured passive behavioral data can be used to detect subtle functional differences across individuals. These results suggest potential future applications of mobile keystroke dynamics in digital health, such as aiding the early detection of cognitive or motor decline and supporting age-sensitive personalization of interventions. However, given that our study included only healthy participants, the clinical applicability of these approaches requires further validation in clinical populations.

## Data Availability

Data sharing is not applicable to this article, as the data cannot be shared publicly due to restrictions imposed by the ethics approval obtained from the institutional review board.

## Abbreviations

AI: artificial intelligence
HT: hold time
LSTM: long short-term memory
MAE: mean absolute error
MLP: multilayer perceptron
PPL: press-to-press latency
XGBoost: extreme gradient boosting
